# Low Abundance of Methanotrophs in Sediments of Shallow Boreal Coastal Zones With High Water Methane Concentrations

**DOI:** 10.3389/fmicb.2020.01536

**Published:** 2020-07-07

**Authors:** Elias Broman, Xiaole Sun, Christian Stranne, Marco G. Salgado, Stefano Bonaglia, Marc Geibel, Martin Jakobsson, Alf Norkko, Christoph Humborg, Francisco J. A. Nascimento

**Affiliations:** ^1^Department of Ecology, Environment and Plant Sciences, Stockholm University, Stockholm, Sweden; ^2^Baltic Sea Centre, Stockholm University, Stockholm, Sweden; ^3^Department of Geological Sciences, Stockholm University, Stockholm, Sweden; ^4^Bolin Centre for Climate Research, Stockholm University, Stockholm, Sweden; ^5^Department of Biology, University of Southern Denmark, Odense, Denmark; ^6^Tvärminne Zoological Station, University of Helsinki, Hanko, Finland

**Keywords:** methane, methanotroph, sediment, DNA, RNA, coast, marine, ebullition

## Abstract

Coastal zones are transitional areas between land and sea where large amounts of organic and inorganic carbon compounds are recycled by microbes. Especially shallow zones near land have been shown to be the main source for oceanic methane (CH_4_) emissions. Water depth has been predicted as the best explanatory variable, which is related to CH_4_ ebullition, but exactly how sediment methanotrophs mediates these emissions along water depth is unknown. Here, we investigated the relative abundance and RNA transcripts attributed to methane oxidation proteins of aerobic methanotrophs in the sediment of shallow coastal zones with high CH_4_ concentrations within a depth gradient from 10–45 m. Field sampling consisted of collecting sediment (top 0–2 cm layer) from eight stations along this depth gradient in the coastal Baltic Sea. The relative abundance and RNA transcripts attributed to the CH_4_ oxidizing protein (pMMO; particulate methane monooxygenase) of the dominant methanotroph Methylococcales was significantly higher in deeper costal offshore areas (36–45 m water depth) compared to adjacent shallow zones (10–28 m). This was in accordance with the shallow zones having higher CH_4_ concentrations in the surface water, as well as more CH_4_ seeps from the sediment. Furthermore, our findings indicate that the low prevalence of Methylococcales and RNA transcripts attributed to pMMO was restrained to the euphotic zone (indicated by Photosynthetically active radiation (PAR) data, photosynthesis proteins, and 18S rRNA data of benthic diatoms). This was also indicated by a positive relationship between water depth and the relative abundance of Methylococcales and pMMO. How these processes are affected by light availability requires further studies. CH_4_ ebullition potentially bypasses aerobic methanotrophs in shallow coastal areas, reducing CH_4_ availability and limiting their growth. Such mechanism could help explain their reduced relative abundance and related RNA transcripts for pMMO. These findings can partly explain the difference in CH_4_ concentrations between shallow and deep coastal areas, and the relationship between CH_4_ concentrations and water depth.

## Introduction

Coastal zones are transitional areas between land and sea where microbes in the water and sediment cycle large amounts of organic and inorganic carbon compounds (Smith and Hollibaugh, 1993). Such zones have recently been shown to be the main source for oceanic methane (CH_4_) emissions (Weber et al., 2019). CH_4_ is a potent greenhouse gas that has increased ∼2.5 times in the atmosphere since the industrial revolution (Stocker et al., 2013), and is today at ∼1.85 ppm (Nisbet et al., 2019), and contributes to approximately ∼20% of tropospheric radiative forcing (Kirschke et al., 2013; Etminan et al., 2016). Furthermore, the annual atmospheric CH_4_ concentration measured during the years 2014–2017 was record high since the 1980s (Nisbet et al., 2019). The majority of CH_4_ emissions are derived from human activities (∼60%) such as livestock (Lassey, 2007), rice paddies (Neue, 1997; Schimel, 2000), hydropower dams (Giles, 2006), and waste management (Dlugokencky Edward et al., 2011). However, natural aquatic systems such as inland waters are reported to contribute a significant portion to CH_4_ emissions (30% or more) (Dlugokencky Edward et al., 2011; Borges et al., 2016; Saunois et al., 2016). In marine ecosystems, coastal zones have the highest contribution to global CH_4_ emissions (Iversen, 1996; Weber et al., 2019), with shallow inshore waters closer to land being estimated to have an annual CH_4_ emission 370 times higher compared to that in the open ocean (Bange, 2006; Osudar et al., 2015; Borges et al., 2016). Globally, shallow water depths in coastal zones are linked to higher CH_4_ emissions (Weber et al., 2019), but environmental predictors have been unable to explain this relationship (Weber et al., 2019). It is therefore possible that biological mechanisms are partly able to explain the discrepancy between coastal shallow and deeper areas. However, this has not been fully investigated and would help to increase the understanding of the controls of CH_4_ cycling in coastal areas.

The cycling of CH_4_ in natural aquatic ecosystems is driven by microbial consumption and production (Bridgham et al., 2013). In brief, the majority of CH_4_ is produced in anoxic zones in sediments as a result of the reduction of e.g., CO_2_, acetate, or methanol by anaerobic methanogenic archaea (Enzmann et al., 2018). Large parts of the produced CH_4_ diffuses upward in the sediment and is oxidized to CO_2_ by anaerobic methanotrophic archaea (ANME) (Knittel and Boetius, 2009), anaerobic methanotrophs (Ettwig et al., 2010), and eventually by aerobic methanotrophs in the oxic sediment surface or the water column (Bowman, 2016). These aerobic methanotrophs thrive on produced CH_4_, and have traditionally been divided into two types: Type I belonging to the Gammaproteobacteria order Methylococcales (Orata et al., 2018); and Type II belonging to the Alphaproteobacteria families Methylocystaceae and Beijerinckiaceae (Kalyuzhnaya et al., 2019). Both types use the enzyme methane monooxygenase (MMO) to oxidize CH_4_, and are able to utilize either the particulate form (pMMO, i.e., bound to the intracellular membrane) and/or the soluble form (sMMO, i.e., enzyme complex in the cytoplasma) (Kalyuzhnaya et al., 2019). In addition to Proteobacteria, the phylum Verrucomicrobia has been found to contain thermophilic aerobic methanotrophs (belonging to the family Methylacidiphilaceae) (Erikstad and Birkeland, 2015). The importance of methanotrophs to limit CH_4_ emission has previously been shown, e.g., Bornemann et al. (2016) used pMMO primers (subunit A, *pmoA*) and clone-libraries to identify methanotrophs (taxonomic order Methylococcales) in the pelagic area of Lake Constance, and found that these bacteria contributed substantially to CH_4_ removal in the bottom water directly above the sediment surface. Bacterial members belonging to the order Methylococcales are ubiquitous (Smith et al., 2018), and metagenome plus metatranscriptome analysis have shown that they dominate aerobic CH_4_ oxidation in wetland soil (Smith et al., 2018), and are important in removing CH_4_ escaping from benthic CH_4_ seeps (Taubert et al., 2019). Methanotrophs are therefore essential key players in regulating CH_4_ emission to the atmosphere from aquatic environments. Although methanotrophs play a key role in CH_4_ cycling and emission to the atmosphere, it is still not fully understood what environmental factors control these populations in marine sediments.

Main factors shown to control methanotrophy include CH_4_ and oxygen availability (King and Blackburn, 1996), and differences in adaptation among methanotrophs have been shown as a response to varying pH, salinity, and oxygen concentration (Knief, 2015). Laboratory studies have also shown that methanotrophs and their activity are stimulated when other heterotrophic bacteria are present (Ho et al., 2014; Veraart et al., 2018). Ammonium (NH_4_^+^) and CH_4_ can be oxidized by both ammonia oxidizing bacteria and methanotrophs, although methanotrophs oxidize CH_4_ more efficiently and vice versa (Bodelier and Frenzel, 1999). High concentrations of NH_4_^+^ have, thus, been reported to have an inhibitory effect on methanotrophic activity (Bédard and Knowles, 1989; He et al., 2017). It has been reported that when NH_4_^+^ has a 30 times higher concentration than CH_4_, methanotrophy is effectively inhibited (Van Der Nat et al., 1997), and potentially this can occur in oxic sediment where methane concentrations are low. Additionally, controlled experimental studies have investigated the role of light availability in mediating methanotrophic activity, but showed contrasting results with both inhibition (Dumestre et al., 1999; Murase and Sugimoto, 2005) and stimulation being reported (Savvichev et al., 2019). Despite this, there is a knowledge gap on the underlying reasons as to why shallow coastal areas have higher CH_4_ emissions. It has been suggested that shallow areas have well-mixed waters where CH_4_ can reach the surface waters easily, and bubbles from CH_4_ seeps in the seafloor can quickly escape to the atmosphere (Borges et al., 2016). However, what role CH_4_ oxidation has in regulating such emissions in these shallow coastal areas and what environmental factors determine CH_4_ oxidizer activity is unknown. Such knowledge is critical to our understanding of the contribution of coastal ecosystems to global CH_4_ budgets.

The aim of the study was to investigate and elucidate why CH_4_ concentrations are higher in shallow inshore coastal water compared to adjacent deeper offshore water. We tested the following hypotheses: (1) the relative abundance of sediment methanotrophs is higher in shallow inshore areas where previous studies have found high concentrations of CH_4_ in the water (possibly favoring growth of methanotrophs); (2) the number of RNA transcripts attributed to MMO (a proxy for CH_4_ oxidation) is higher in shallow inshore sediments; and (3) bottom water oxygen and pore water NH_4_^+^ concentrations regulate the number of RNA transcripts attributed to MMO in the sampled sediments.

## Materials and Methods

### Sediment Collection and Water Column Profiles

**FIGURE 1:**
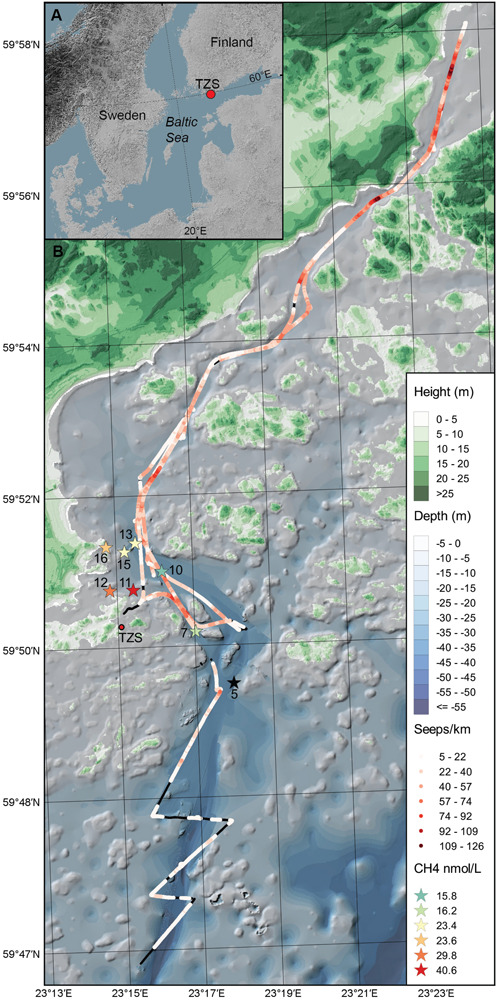
**(A)** The map shows the Baltic Sea and the location of the study area in the Western Gulf of Finland. **(B)** Sediment was collected (top 0–2 cm surface) during June 2017 and September 2018 from the eight sampling stations (denoted as stars with station number, *n* = 3 sediment cores per station) in the Storfjärden bay, close to the Tvärminne Zoological Station (TZS). The red colored gradient of the stars shows the CH_4_ concentration in the surface water for each station (0.5–1.0 m, for station five no CH_4_ data was collected). The red-colored gradient line on the map shows the cruise track and number of CH_4_ seeps km^–1^ observed with acoustic data in the bottom water above the sediment. Black lines on the cruise track denotes no CH_4_ seeps observed. The blue gradient denote water depth (m) and the green gradient terrestrial land height (m).

Sediment slices (top 0–2 cm) were collected along coastal gradients (0–4 km, 10–45 m water depth) on board R/V Electra in Storfjärden bay close to the Tvärminne Zoological Station (TZS), Tvärminne, Finland (Figure 1A). Triplicate sediment cores were collected from each station during June 2017 and September 2018 (Table 1). All samples were collected using a GEMAX twin gravity corer in combination with acrylic tubes (height: 80 cm, inner diameter: 80 mm). From each core the top 0–2 cm sediment surface layer was sliced into either plastic bags (freezer bags, 2017 sampling) or autoclaved 215 ml polypropylene containers (Noax Lab; 2018 sampling). June 2017 sediment was collected for DNA extraction from eight stations (due to logistical reasons RNA was not collected), while September 2018 sediment was collected from seven stations for DNA and RNA extraction (*n* = 3 per station for both years). The stations were divided into four offshore sites (stations 5, 7, 10, 13; 36–45 m deep) and four inshore sites (stations 11, 12, 15, 16; 10–28 m deep) (Figure 1B and Table 1). For the 2018 sampling sediment slices from each station was aseptically homogenized inside the containers and 2 ml sediment transferred into 2 ml cryogenic tubes (VWR), flash frozen in liquid nitrogen, and stored at −80°C at TZS. All collected sediment for DNA was stored at −20°C on the boat until transferred into a cooling box filled with ice bars and transported to Stockholm University (∼1 h). The flash frozen 2 ml sediment for RNA were transported from TZS to Stockholm University on dry ice, and stored again at −80°C until RNA extraction. The DNA data was used to investigate methanotrophic microorganisms in the sediment, while RNA transcript data was used to identify methanotrophs and the transcription of genes coding for methane monooxygenase.

**TABLE 1:** List of the station numbers (“off” denotes offshore stations (5, 7, 10, 13), and inshore stations are denoted solely by their station number 11, 12, 15, 16), the number of sediment cores collected and sliced (0–2 cm surface) for DNA/RNA extraction or pore water NH_4_^+^ analyses, sampling dates during June 2017 and September 2018, latitude, longitude, and water column depth.

**Station**	**DNA/RNA extraction (*n*)**	**NH_4_^+^ analyses (*n*)**	**2017 date**	**2018 date**	**Lat. (dd)**	**Long. (dd)**	**Depth (m)**
Off-5	3	–	June 4	–	59.8319	23.29566	45
Off-7	3	3	June 4	September 23	59.8430	23.28035	37
Off-10	3	3	June 4	September 20	59.8559	23.26695	36
11	3	3	June 4	September 20	59.8521	23.25475	18
12	3	3	June 4	September 22	59.8521	23.24495	10
Off-13	3	3	June 4	September 22	59.8620	23.25615	40
15	3	3	June 5	September 20	59.8602	23.25155	28
16	3	3	June 5	September 22	59.8613	23.24387	10

CTD profiles of PAR light and oxygen concentrations (SEA-Bird SBE 911 plus) were collected in the study area from 12 locations during the 2018 sampling campaign between September 19–23. This information was used to infer how light and oxygen availability might have affected methanotrophs.

### Real-Time Measurement of Methane in the Surface Water

In September 2018 CH_4_ concentrations in the surface water at a 0.5–1.0 m water depth were measured *in situ* using a Water Equilibration Gas Analyzer System (WEGAS). A full method description along with the results are presented in Humborg et al. (2019). In brief, circulation pumps equipped to a seawater inlet transfer seawater into an equilibrator with showerhead. The gas is transferred through a gas handling system, and is analyzed for CH_4_ concentrations by a cavity ring-down spectrometer gas analyzer (Picarro G2131-i). This system also tracked temperature and salinity as long as R/V Electra was cruising. Salinity, temperature, and CH_4_ for September 2018 have been measured and is available in Humborg et al. (2019). However, data from the specific stations presented here have not been reported.

### Acoustic Data of Methane Seeps From the Sediment

Acoustic data were collected during the September 2018 sampling campaign (Figure 1B). The acoustic data were collected with a Simrad EK80 wide band transceiver, transmitting through a hull mounted Simrad ES70-7C split beam transducer with a center frequency of 70 kHz. Position and attitude information were provided to the echo sounder as an integrated solution by a Seapath 330 + GPS/GLONASS navigation and motion reference system. The Seapath 330 + received real-time kinematic (RTK) positional corrections from the Finnish system of stations *FinnRef*^1^, resulting in horizontal accuracies better than ±5 cm and slightly coarser vertical accuracies. The acoustic EK80 dataset was match filtered with an ideal replica signal using a MATLAB software package provided by the system manufacturer (Lars Anderson, personal communication). Seeps were defined as either trains of bubbles or bubble plumes (many bubbles overlapping in vertical structures) and were identified through visual inspection of the processed acoustic data. Ebullition from sediments has been observed in the study area and reported in Humborg et al. (2019). Here we present in addition high resolution acoustic data on: (1) the number of seeps and (2) the relation of seeps to water depth in the study area. The number of seeps per km was derived by applying a running average with a window size of 0.2 km along the cruise track. For calculations of seeps per km as a function of depth, the total ship track (about 65 km) as well as the number of observed seeps (in total 1975 observations) were divided into 1 m seafloor depth bins ranging from 5 to 60 m. Depths along the survey track were derived from the EK80 bottom returns. The number of seeps in each depth bin was then divided by the track length within each depth bin. Note that the tendency of decreasing number of seeps per km with increasing depth becomes significantly stronger if accounting for the footprint of the echo sounder beam (Supplementary Figure S1). This is because the beam footprint increases with depth. While this should provide a more accurate picture in theory, there might be issues with overlapping seeps (multiple seeps being counted as one), and the actual seep distribution might be somewhere in between. The seafloor bathymetry in the vicinity of the EK80 survey track, between about 59°47′N and 59°51′N, was previously mapped using R/V Electra’s Kongsberg EM2040 0.4 × 0.7, 200–400 kHz, multibeam echo sounder (Jakobsson et al., 2020). Figure 1B displays a shaded relief of the acquired multibeam bathymetry.

### Ammonium Analyses

Sediment frozen at −20°C sampled during the September 2018 campaign was thawed and 20 ml of sediment was transferred into 50 ml centrifuge tubes (Sarstedt). Pore water was extracted by centrifugation at 2200 × *g* at 9°C followed by filtration of 10 ml supernatant through a 0.45 μm polyethersulfone membrane filter (Filtropur S 0.45, Sarstedt). The pore water was then stored at −20°C until NH_4_^+^ analyses. Pore water samples were analyzed colorimetrically (Multiskan GO spectrophotometer, Thermo Scientific) for ammonium concentrations and the modified salicylate-hypochlorite method of Bower and Holm-Hansen (1980) was used.

### Nucleic Acids Extraction and Sequencing

Approximately 10 g and 2 g of sediment were used to extract DNA and RNA using the DNeasy PowerMax Soil kit (QIAGEN) and RNeasy PowerSoil kit (QIAGEN) kits, respectively. DNase treatment was conducted on extracted RNA by using the TURBO DNA-free kit (Invitrogen). This was followed by ribosomal RNA depletion with the RiboMinus Transcriptome Isolation Kit (ThermoFisher Scientific). A 2100 Bioanalyzer (Agilent) was used to confirm that the RNA samples were free of DNA contamination. Library preparation of DNA and RNA samples were prepared with the ThruPLEX DNA-seq (Rubicon Genomics) and TruSeq RNA Library Prep v2 (without the poly-A selection step, Illumina) kits, respectively. The Illumina NovaSeq6000 platform was used to sequence DNA and RNA, with one S2 and S4 lane used for the DNA and RNA samples (a paired-end 2 × 150 bp setup), respectively. All samples were sequenced at the Science for Life Laboratory, Stockholm. The sequences are available in the NCBI BioProject repositories, PRJNA541421 (DNA) and PRJNA54 1422 (RNA).

### Quality Trimming of Sequences

SeqPrep 1.2 (St John, 2011) was used to remove Illumina adapters from the sequences. This was followed by removal of any leftover PhiX sequences by mapping the reads against the PhiX genome (NCBI Reference Sequence: NC_001422.1) using bowtie2 2.3.4.3 (Langmead and Salzberg, 2012). Trimmomatic 0.36 (Bolger et al., 2014) was used to quality trim reads with the following parameters: LEADING:20 TRAILING:20 MINLEN:50. Final quality of the trimmed reads were checked with FastQC 0.11.5 (Andrews, 2010) and MultiQC 1.7 (Ewels et al., 2016). See Supplementary Data S1 for full details of sequence counts before and after quality trimming, average read lengths, and extracted 16S rRNA gene sequences etc.

### Taxonomic Annotation

All metagenomic DNA sequences and SSU rRNA from the DNA and RNA sequence data were taxonomically classified. SSU rRNA data was extracted with SortMeRNA 2.1b (Kopylova et al., 2012) followed by annotation using Kraken2 2.0.7 (Wood et al., 2019). Kraken2 was run with a paired-end setup against the NCBI RefSeq genome database (database downloaded 1 March 2019) for all metagenome DNA sequences, while SSU rRNA gene data was run against the small-subunit SILVA (Quast et al., 2013) (for prokaryotic data, database downloaded 1 March 2019) and NCBI NT (for better classification of eukaryotic 18S rRNA gene sequences; database downloaded 12 March 2019). To more accurately estimate the relative abundance of the classified prokaryotic taxonomy Bracken 2.5 (Lu et al., 2017) was used on the Kraken2 reports (run with default parameters on the genus level and a count threshold of 10). The final Kraken2 reports were then combined into a biom-format file using the python package kraken-biom 1.0.1 (with the following setup: -fmt hdf5 -max D -min G). The python package biom-format 2.1.7 (Mcdonald et al., 2012) was then used to convert the biom-table to a text table. In the taxonomy table chloroplast sequences were removed, and data was normalized as relative abundance (%) and analyzed in the software Explicet 2.10.5 (Robertson et al., 2013).

### Protein Classification of Functional Genes and RNA Transcripts

Paired-end DNA and RNA sequences were merged using PEAR 0.9.10 (Zhang et al., 2014), and non-rRNA sequences were extracted using SortMeRNA/2.1b. Protein annotation was conducted by aligning sequences against the NCBI NR database (*e*-value threshold <0.001, database downloaded 2 April 2 2019) using Diamond 0.9.10 (Buchfink et al., 2015) in conjunction with BLASTX (Altschul et al., 1990). MEGAN 6.15.2 (Huson and Mitra, 2012) was used to analyze the taxonomy and protein classification of the output diamond files using default LCA parameters and software supplied databases (taxonomy: prot_acc2tax-Nov2018X1.abin, and InterPro protein database: acc2interpro-June2018X.abin). To distinguish between AMO and pMMO sequences reads classified against the AMO/pMMO protein family was extracted from MEGAN. The extracted sequences were classified against the protein UniProtKB/Swiss-Prot database (2019 July version) and the taxonomy NCBI NT database (date: 2020-05-27) using BLASTX 2.7.1 + with an *e*-value threshold of 0.001. Data was normalized among samples as counts per million sequences (CPM; relative proportion × 1,000,000).

In addition to the assembly-free approach of the RNA transcripts, the metagenomic quality trimmed DNA sequences were used to construct a co-assembly with MEGAHIT 1.1.2 with default settings (Li et al., 2016). This yielded an assembly with 33,857,159 contigs (average contig length: 736). Prodigal 2.63 (Hyatt et al., 2010) was used with default settings to predict genes and proteins in the assembly. The predicted genes were classified using BLASTX (*e*-value threshold <0.001) against the UniProtKB/Swiss-Prot database, and genes classified to code for *pmoA* and *pmoB* were extracted. The *pmoAB* genes were delimited to read lengths >500 bp, and the final list of genes was manually checked against the UniProtKB/Swiss-Prot database to ensure they code for particulate methane monooxygenase. The *pmoAB* sequences were used as a reference that the RNA sequence data was mapped against. Bowtie2 2.3.4.3 (Langmead and Salzberg, 2012) was used to build a reference, followed by mapping of the RNA sequences to the *pmoAB* reference. Samtools 1.9 (Li et al., 2009) was used to extract the amount of counts mapped (both R1 and R2 pairs required to map), using the following parameters: samtools view -c -f 1 -F 12. The final count data was normalized for each sample as CPM values based on each respective metagenome library size. A full list of contig details from the metagenome assembly and prodigal predicted *pmoAB* genes is available in Supplementary Data S2.

### Quantitative Reverse Transcription PCR (RT-qPCR)

For each DNase treated RNA sample a total of 700 ng were reverse transcribed with random primers using the AccuScript High Fidelity 1st Strand cDNA Synthesis kit (Agilent). The cDNA samples were diluted × 10 and 2 μl were used as a template in 5 μl qPCR reactions. Reactions were prepared with the LightCycler 480 SYBR Green I Master kit (Roche) using 300 nM of each primer, and qPCR was then conducted using an Eco Real Time PCR system (Illumina). The 16S rRNA primer pair 515F and 805R (Herlemann et al., 2011; Parada et al., 2016) were used to amplify 16S rRNA genes reverse transcribed to cDNA as an internal normalizer. The RT-qPCR conditions were: an initial denaturation (95°C, 10 min) followed by a single step for annealing and extension at 64°C, 30 s. Primers targeting *pmoA* genes reverse transcribed to cDNA were designed based on metagenome assembled *pmoA* genes (Supplementary Data S2). Primers were designed using Primer3 at NCBI Primer-Blast server and the primer sequences are listed in Supplementary Data S3. Degenerate *pmoA* primers (Fwd: GAGYGCATCTCAATCAGCTGTACG, Rv: GTCCAGAAATCCCAGTCACCRC) targeting a 153 bp long fragment were used for RT-qPCR with an initial denaturation step (95°C, 10 min) followed by annealing (60°C, 30 s) and extension (72°C, 5 s) (additional non-degenerate primers are listed in Supplementary Data S3). To confirm that there was no genomic DNA and primer dimer contamination the inclusion of a water template, an RT-minus run, and analyzes of melting dissociations curves combined with gel electrophoresis were included in the analysis. The RT-qPCR *pmoA* results were normalized against those of 16S rRNA using the 2^–Δ^ ^Δ^ ^*Ct*^ method by Livak and Schmittgen (2001).

### Statistics

Alpha diversity (Shannon’s H) was conducted using the 2018 16 rRNA gene data (for all taxonomic classifications, as shown in Supplementary Data S4) in the software Explicet after sub-sampling to the lowest sample size (8753 counts) and bootstrap × 100. Non-metric multidimensional scaling (NMDs, based on the Bray-Curtis dissimilarity index of the relative abundances) and Principal Component Analysis (PCA) of the dataset was analyzed in the software past 3.22 (Hammer et al., 2001). Shapiro–Wilk tests were used to test for normal distribution of the taxonomy data, and SPSS 26 was used to test for differences among stations using non-parametric Kruskal–Wallis tests or One-Way ANOVAs with *post hoc* Tukey HSD tests for parametric data. Correlations between variables were conducted with Spearman correlations using data from all stations (two-tailed). Correlation between CH_4_ nM and the relative abundance of Methylococcales in the sediment was done by using the same nM value for CH_4_ for each replicate sediment core, as CH_4_ was measured in the water surface. Acoustic data of CH_4_ seeps was correlated with water depth using Pearson correlation in the software MATLAB 2017a. Differences in metabolic functions (InterPro classifications of RNA transcripts data) were tested with the R package edgeR 3.24.3 (Robinson et al., 2010). In more detail, the perl script “run_DE_analysis.pl” supplied with Trinity 2.8.2 (Haas et al., 2013) was used to run the analysis. The script inputs raw read data, normalize read counts, and analyze differential gene expression using edgeR. Statistic significances were indicated by false discovery rate (*FDR*) values <0.05. To test for differences in DNA sequencing counts of Methylococcales between offshore and inshore stations in 2018, DESeq2 analyses was conducted with the R package DESeq2 1.26 using default settings (Love et al., 2014). DESeq2 does not normalize data proportionally, but uses size factors determined from the median-of-ratios method adjusting for sequencing depth. Counts for Methylococcales 16S rRNA gene sequences, all other 16S rRNA gene sequences, and the total library size of the DNA metagenome data was used as an input, and the difference between inshore and offshore stations was tested. The DESeq2 output was plotted using the ggplot2 package in R (Wickham, 2016).

## Results

### Water Column Parameters

Salinity ranged from 6.5–7.0 ppt with higher salinity in the bottom water and did not differ between inshore and offshore stations. Temperature ranged from 3.4–8.9°C (2017 early June) and 6.02–15.82°C (2018 late September), with higher temperatures in the surface water. CTD profiles taken in September 2018 of the water column from twelve locations inside the study area showed that photosynthetically active radiation (PAR) light reached a water depth of 28 m at sites <30 m water depth, and the inshore stations would therefore have been illuminated (Supplementary Figure S2). The bottom water was oxic with oxygen concentrations between 7.6 and 8.6 ml/l in the study area as measured in September 2018 (Supplementary Figure S2).

### CH_4_ Concentrations in the Surface Water

CH_4_ concentrations in the surface water measured in September 2018 were higher in the inshore shallow stations close to land (23.4–40.6 nM, *n* = 4 stations) compared to the offshore areas (16.2–23.4 nM; *n* = 3 stations, Figure 1B and Supplementary Table S1).

### Alpha and Beta Diversity

In the top 0–2 cm sediment layer prokaryotic alpha diversity ranged between 5.3 and 6.2 (Shannon’s H, 16S rRNA gene 2018 data) with no difference between inshore and offshore stations (Kruskal–Wallis test). NMDs of Bray-Curtis beta diversity showed that the offshore stations 7, 10, and 13 clustered differently compared to inshore stations 11, 12, 16, and 15 (PERMANOVA 9999 permutations, *F* = 8.5, *P* < 0.01 for the whole model; Supplementary Figure S3). A full list of the prokaryotic classifications and sequence counts is available in Supplementary Data S4.

### Methanotrophs in the Sediments

**FIGURE 2:**
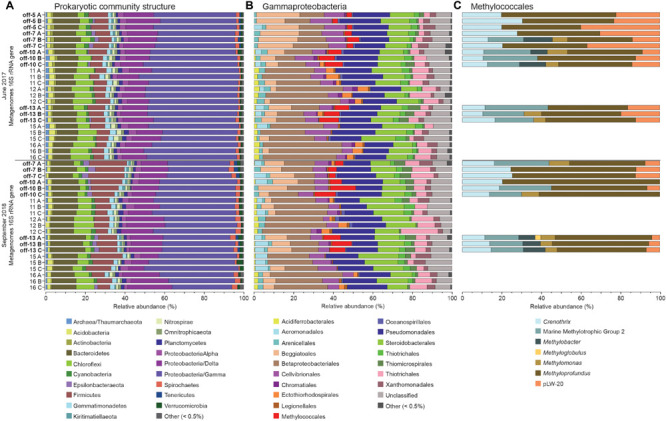
The stacked bars show relative abundance (*x*-axis%) of metagenome extracted 16S rRNA gene sequences: **(A)** prokaryotic phyla and Proteobacteria classes, **(B)** Gammaproteobacteria orders, and **(C)** Methylococcales genera (only detected in offshore stations). For **(A,B)** the dataset was delimited to taxonomic groups >0.5% (average of all samples). The *y*-axis shows the sampling year, and station names and replicate samples indicated by letters A, B, and C. Offshore stations are indicated on the *y*-axis with the label “off”.

**FIGURE 3:**
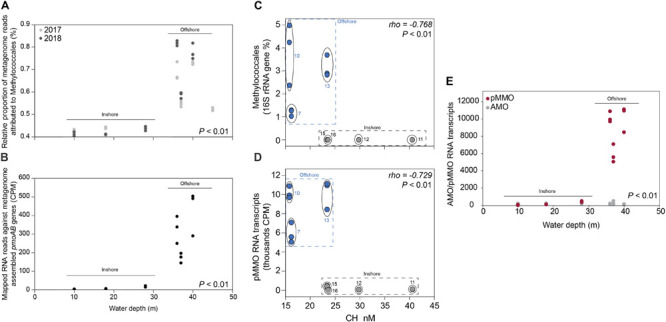
**(A)** The relative proportion of reads (*y*-axis%) attributed to the methanotrophic Gammaproteobacteria order Methylococcales compared to the whole prokaryotic community. The data was based on all metagenomic sequences classified against the NCBI RefSeq genome database. The *x*-axis shows the water depth (m), each circle represents one sediment core, and the colors denotes the sampling year (light gray as 2017, and dark gray as 2018). The water depth for the inshore stations are: 10 m (stations 12 and 16), 18 m (11), 28 m (15); and offshore stations: 36 m (10), 37 m (7), 40 m (13), and 45 m (5). **(B)** Mapped RNA reads (2018 RNA data) against metagenome assembled *pmoAB* genes (>500 bp long, 2018 DNA data). The data shows normalized sequence counts (counts per million sequences; CPM values). The *x*-axis shows the water depth (m), and each circle represents one sediment core. **(C)** The relative abundance of the methanotrophic Gammaproteobacteria order Methylococcales in the 0–2 cm sediment surface (*y*-axis, *n* = 3 per station) and measured CH_4_ in the water column (*x*-axis, *n* = 1 per station). The relative abundance of Methylococcales shown is based 16S rRNA gene sequences extracted from the 2018 DNA data, while CH_4_ was measured in the water surface (0.5–1.0 m water depth). **(D)** RNA transcripts classified as pMMO against the UniProtKB/Swiss-Prot database (based on classifying all paired-end merged RNA reads). Values shown are normalized sequence counts (CPM). Blue filled circles denote stations further away from the coast, and the gray circles show the sampling stations for each cluster of data points. **(E)** RNA sequences annotated to the InterPro AMO/pMMO family was classified against the UniProtKB/Swiss-Prot database and compared to the water depth (m) of the stations (*x*-axis). CPM values shown are based on all proteins classified against the InterPro database (*y*-axis). Each circle in the graph denotes RNA transcripts derived from the 0–2 sediment surface from individual sediment cores. Dark red circles denote pMMO and light gray circles denote AMO. The *P*-values indicate the statistical significance (Kruskal–Wallis tests) for Methylococcales, *pmoAB*, and pMMO between inshore and offshore **(A,B,E)**, and alongside *rho* values that show results from Spearman correlations **(C,D)**.

Gammaproteobacteria had the highest relative abundance of the prokaryotic community in the 0–2 cm sediment surface when comparing phyla and Proteobacteria classes between stations (Figure 2A). In the metagenome 16 rRNA gene data the relative abundance of Gammaproteobacteria ranged between 30–51% for all stations (Figure 2A). The relative abundance of the Type I CH4 oxidizing taxonomic order Methylococcales was significantly higher in stations located offshore (Kruskal–Wallis tests, df = 1, *H* = 19.7, *P* < 0.01 (2017 16S rRNA gene data), df = 1, *H* = 18.1, *P* < 0.01 (2018 16S rRNA gene data; Figure 2B). A closer look at the Methylococcales order showed that CH_4_ oxidizers was composed of bacteria belonging to the family Methylomonaceae with majority of sequences (up to 79.7%) classified to the genus Methyloprofundus (Figure 2C). In the 16S rRNA gene data Methylococcales had a relative abundance up to 4.98% of the whole microbial community (Supplementary Data S4). Furthermore, taxonomic classification of all metagenomic sequences against the NCBI RefSeq genome database (Supplementary Data S5) showed that Methylococcales was attributed a higher relative proportion of reads in the offshore stations compared to the inshore stations (Kruskal–Wallis tests, 2017 DNA data, *H* = 17.4, *P* < 0.01; 2018 DNA data, df = 1, *H* = 14.8, *P* < 0.01; Figure 3A). Similarly, based on mapping RNA reads against metagenome assembled *pmoAB* genes (Supplementary Data S2), more RNA reads were mapped in the offshore stations compared to the inshore stations (Kruskal–Wallis test, df = 1, *H* = 14.8, *P* < 0.01; Figure 3B and Supplementary Table S1). To test that the higher relative abundance of methanotrophs was not an effect of sequencing depth, we compared the count data of Methylococcales 16S rRNA gene sequences with all other 16S rRNA gene sequences, and with the total library sizes with DESeq2. The results showed that in the offshore stations the Methylococcales 16S rRNA gene counts had a log2 fold change of 10.5 (for June 2017) and 10.9 (September 2018) compared to the inshore stations. In contrast, the counts for other 16S rRNA gene sequences and the total library size had both a log2 fold change of 0.1 for both years (Supplementary Figure S4).

### RNA Transcripts Attributed to Methane Oxidation

**FIGURE 4:**
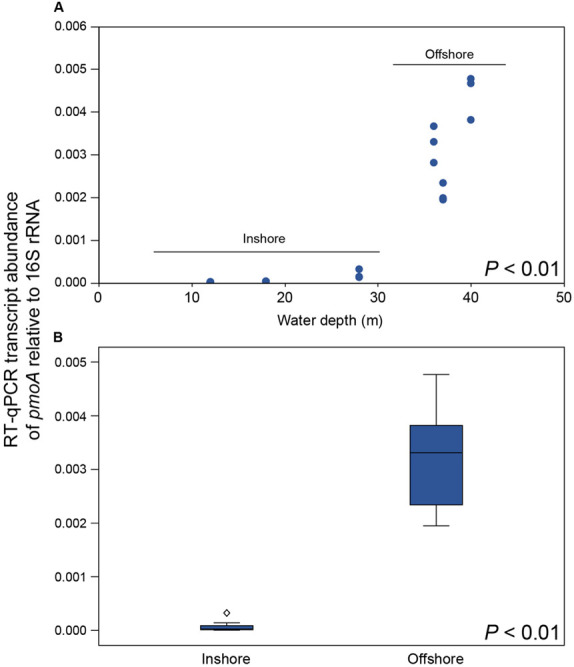
**(A)** RT-qPCR results showing the number of *pmoA* RNA transcripts relative to 16S rRNA for each station along the water depth gradient (*y*-axis shows NRQ; normalized relative quantification). **(B)** Boxplot graph showing the inshore stations compared to the offshore stations. The diamond symbol denotes an outlier 3 or more box lengths from the median. The *P*-values indicate the statistical significance (One-Way ANOVA) between inshore and offshore.

CH_4_ concentrations in the 0.5–1.0 m water surface showed a negative relationship with the 16S rRNA gene relative abundance of Methylococcales in the sediment, with lower CH_4_ concentrations in the offshore stations (Figure 3C) where the metagenome data indicated more RNA transcripts attributed to *pmoAB*. Furthermore, CH_4_ concentrations measured in the surface water during September 2018 correlated negatively with the relative abundance of Methylococcales for the same-year 16S rRNA gene data (rho = −0.768, *P* < 0.01, *n* = 21). That Methylococcales was associated with offshore sites further away from the coast was also indicated by positive correlations with water depth (2018, 16S rRNA gene data, rho = 0.818, *P* < 0.01; 2017, 16S rRNA gene data, rho = 0.740, *P* < 0.01). In addition to a higher relative abundance of Methylococcales, RNA transcripts attributed to the protein family AMO/pMMO also correlated negatively with measured concentrations of CH_4_ (based on classifying all paired-end merged RNA sequences, rho = −0.760, *P* < 0.01, *n* = 21; See Supplementary Data S6 for all protein classifications). RNA transcripts attributed to AMO/pMMO were also significantly higher in the offshore stations (*FDR* < 0.05, test between all stations individually; Supplementary Table S1), while functional genes in the metagenome attributed to AMO/pMMO were available at all stations with little difference in CPM values (counts per million sequences) (1429–1652 CPM; Supplementary Table S1), showing that the potential to oxidize CH_4_ was available at all sites. Similarly to the 16S rRNA gene data, the pMMO sequences consisted of Methylococcales and was dominated by the genus Methyloprofundus (Supplementary Figure S5) when aligned against NCBI NT. The soluble form of MMO was not detected in the RNA transcript dataset (Supplementary Data S6), but was present with low CPM values in the metagenome data (Supplementary Data S7). AMO/pMMO sequences were classified against the UniProtKB/Swiss-Prot database to separate AMO and pMMO sequences. The resulting pMMO CPM values correlated negatively with the CH_4_ concentrations in the surface water (rho = −0.726, *P* < 0.01; Figure 3D). Furthermore, there was also a large difference in pMMO CPM values between the offshore stations (8742 ± 2342 CPM, one standard deviation shown) compared to the shallower inshore stations (58 ± 175 CPM, Kruskal–Wallis test, df = 1, *H* = 14.7, *P* < 0.01; Figure 3E). These pMMO sequences were affiliated with the reference species *Methylococcus capsulatus* in the UniProtKB/Swiss-Prot database. That methanotrophy was higher in the offshore stations compared to inshore was also supported with quantitative reverse transcription PCR (RT-qPCR) based on RNA samples and degenerate *pmoA* primers (Supplementary Data S3). The offshore stations had 0.003257 ± 0.001067 NRQ (normalized relative quantification, i.e., *pmoA* RNA transcripts relative to 16S rRNA) compared to the inshore stations with 0.000066 ± 0.000094 NRQ (One-Way ANOVA, *F*_(__1_,_19__)_ = 108.0, *P* < 0.01; Figure 4 and Supplementary Table S1; results from non-degenerate primers are shown in Supplementary Figure S6).

### Methanotrophs and Light

Because light has been indicated to inhibit CH_4_ oxidation we also analyzed the amount of RNA transcripts attributed to proteins in the Gene Ontology (GO) category Photosynthesis (Supplementary Table S1 and Supplementary Data S6). Photosynthesis proteins in the sediment surface had a negative correlation with both the relative abundance of Methylococcales (2018 data, rho = −0.615, *P* < 0.01), and AMO/pMMO enzymes (rho = −0.760, *P* < 0.01, *n* = 21). Photosynthesis proteins were also negatively correlated with water depth (rho = −0.676, *P* < 0.01, *n* = 21; Supplementary Figure S7). Moreover, 18S rRNA data of diatoms showed a higher relative abundance of benthic genera such as *Amphora* and *Nitzschia* in the inshore stations, which provides further indication that these stations were euphotic (Supplementary Figure S8). This in accordance with the PAR data that indicated the inshore areas to be illuminated while offshore bottom zones were in darkness. A full list of proteins can be found in Supplementary Data S6 (RNA) and Supplementary Data S7 (DNA).

### Other Aerobic Methanotrophs

Our results clearly show that Methylococcales were the major methanotroph active in the sediments, while other methanotrophs were found to be absent in the 16S rRNA gene dataset. This absence encompassed the Type II methanotrophic families Methylocystaceae and Beijerinckiaceae (belonging to Alphaproteobacteria), the Verrucomicrobia family Methylacidiphilaceae, and the NC10 phylum known to contain anaerobic methanotrophs (Supplementary Data S1). However, some of these taxa were present when classifying all metagenome sequences against the RefSeq genome database (Supplementary Data S2). It is therefore possible that these organisms were present in our study site but not in high abundance to be detected in the extracted 16S rRNA gene sequences.

### Pore Water Ammonium Concentrations

NH_4_^+^ analyses showed that the pore water concentration of NH_4_^+^ was higher in the offshore stations (308 ± 59 μM) compared to the inshore stations (196 ± 49 μM; One-Way ANOVA, *F*_6_,_14_ = 33.1, *P* < 0.01, with Tukey *post hoc* test between stations, *P* < 0.01; Supplementary Table S1). The 16S rRNA gene dataset for both years 2017 and 2018 showed that aerobic ammonia oxidizing bacteria and archaea were present at all sites and had a significantly higher relative abundance in the inshore sites (inshore: 2017, 6.9 ± 1.5; 2018, 5.4 ± 1.5%, compared to offshore: 2017, 4.2 ± 1.0; 2018, 3.4 ± 1.3%) (Kruskal–Wallis tests, 2017, *H* = 14.9, *P* < 0.01; 2018, *H* = 6.5, *P* = 0.011; Supplementary Figure S9). However, AMO transcripts showed no differences in CPM values between the offshore and inshore stations (196 ± 90 CPM, Kruskal–Wallis test, *H* = 0.4, *P* = 0.83; Figure 3E), suggesting that pore water NH_4_^+^ concentrations did not explain the difference in RNA transcripts attributed to pMMO (i.e., due to inhibition of methanotrophy) between inshore and offshore areas.

### Methanotrophic and Methanogenic Archaea in the Sediment

Archaea had a 0–2% relative abundance in the 0–2 cm sediment (Figure 2), and methanotrophic archaea (ANME) were not detected in the 16S rRNA gene data for any of the years (Supplementary Data S4). Similarly, methanogenic archaea (e.g., Methanobacteria and Methanomicrobia) were not detected in the 16S rRNA gene data (Supplementary Data S4). However, a few sequences affiliated to methanogens were present in the metagenome sequences when classified against the NCBI RefSeq database and methanogenesis in the RNA transcripts data. Potentially some methanogens were present in the sediment in the sediment layers here sampled, but not in enough abundance to be detected in the extracted 16S rRNA gene sequences.

### Methane Escape From the Sediment

**FIGURE 5:**
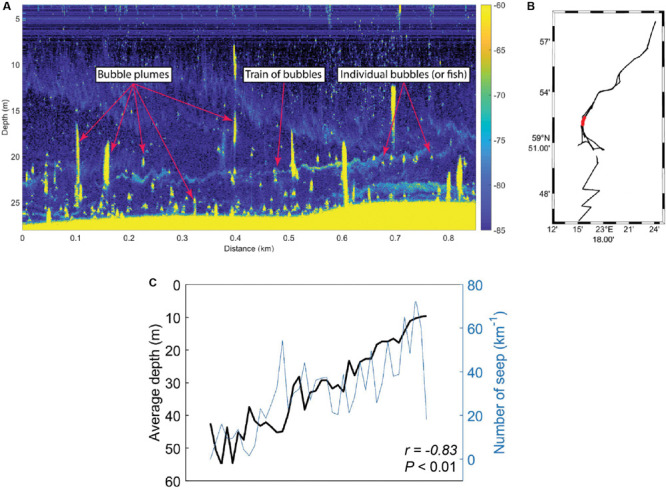
Onboard the research vessel acoustic data (EK80 wide band transceiver) was collected from the southern, central, and northern Storfjärden bay. **(A)** Example of echogram with seeps from the seafloor and within the water column. The right-side *y*-axis shows target strength (dB) as a function of horizontal distance (*x*-axis) and water depth (left-side *y*-axis). **(B)** Map of the Storfjärden bay showing the data track from all the acoustic data that were collected (black) and the track shown in the echogram in A (red). **(C)** Water depth (black line, *y*-axis increasing with lower water depth) compared to the number of CH_4_ seeps observed (blue line, *y*-axis increasing with more CH_4_ seeps). The *r* and *P*-value shows the results from the Pearson correlation.

Acoustic data of the seafloor and water column was collected in the study area during September 2018, and CH_4_ seeps from the seafloor were defined as either trains of bubbles or bubble plumes (Figures 5A,B). The results showed that the prevalence of CH_4_ seeps in sediment surface was generally greater in shallow areas compared to deeper areas when taking the entire survey area into account (Figure 5C). Moreover, the amount of CH_4_ seeps km^–1^ was negatively correlated with water depth (Pearson correlation, *r* = −0.83, *P* < 0.01, *n* = 52).

## Discussion

### Methanotrophs in Inshore and Offshore Coastal Zones

**FIGURE 6:**
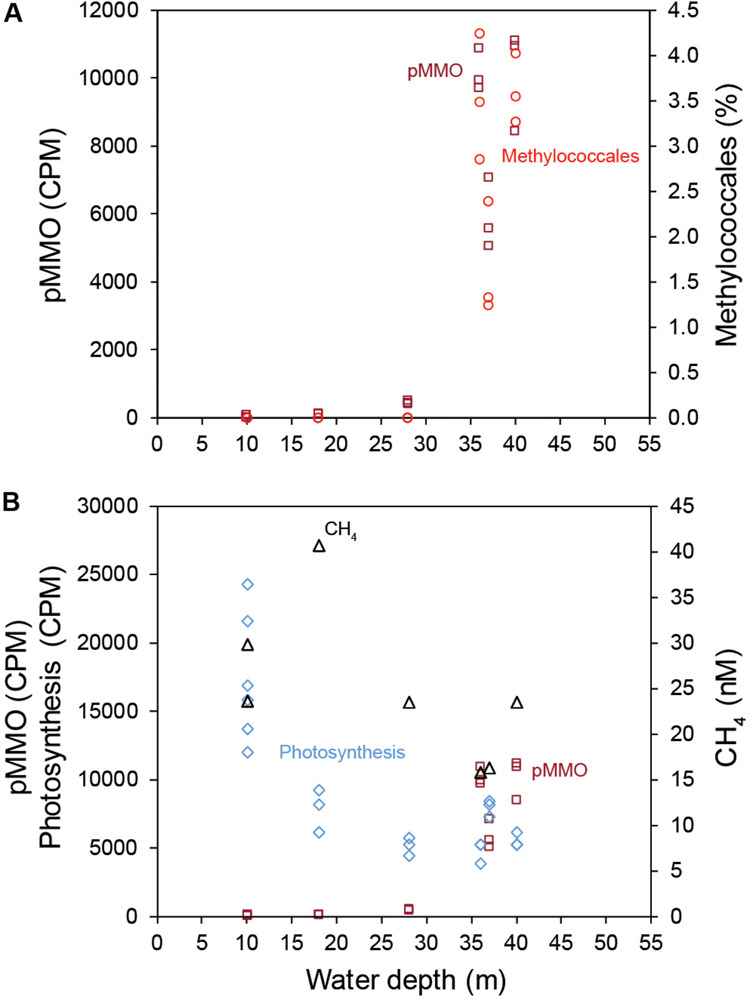
**(A)** The relationship between water depth (*x*-axis), the relative abundance (%) of Methylococcales (16S rRNA gene 2018 data, red), and pMMO transcripts (CPM, dark red). **(B)** The relationship between water depth and CH_4_ concentration in the surface water (black symbols), pMMO transcripts (CPM, dark red), and the sum of transcripts belonging to the GO category photosynthesis (CPM, light blue).

The results presented in this study suggest that the higher CH_4_ concentrations in shallow coastal zones are partly explained by low abundance of methanotrophs and RNA transcripts attributed to pMMO. Furthermore, the shallow areas in the studied bay had more CH_4_ seeps escaping the sediment, and higher CH_4_ concentrations in the surface water. This could partly explain why shallow coastal zones are known to have high CH_4_ concentrations in the water column compared to deeper waters (Bange, 2006; Osudar et al., 2015; Borges et al., 2016). CH_4_ ebullition might bypass aerobic methanotrophy in the inshore areas and contribute to higher CH_4_ concentrations in the surface water. However, it is likely that low methanotrophic activity allows dissolved CH_4_ to escape the sediment surface. Interestingly, we only observed a reduced number of RNA transcripts attributed to pMMO in the inshore shallow areas. These findings were supported by the significantly lower relative abundance of 16S rRNA gene sequences classified as methanotrophs, and lower RNA transcripts attributed to CH_4_ oxidation (pMMO) in the inshore areas (Figure 6A). The higher abundance of RNA-seq *pmoA* transcripts in offshore areas, as analyzed bioinformatically, was further confirmed with a different method using RT-qPCR. The metagenome data showed pMMO to be present at all stations (inshore and offshore), further indicating a decreased activity on CH_4_ oxidation in the inshore areas. This indicates a low relative abundance of aerobic methanotrophs in the inshore areas. However, they could not be detected when the 16S rRNA gene sequences were extracted (but are present when all metagenome sequences were classified against the RefSeq database; Figure 3A). A majority of the Methylococcales sequences were classified to the genus *Methyloprofundus*, an obligate aerobic methanotroph only able to utilize the pMMO pathway (Tavormina, 2015), which was in accordance to our RNA transcript data. The soluble form of MMO was detected in the metagenome data but not in the RNA transcript data. Because pMMO is a copper-containing enzyme, a low ratio of copper-to-biomass initiates expression of sMMO (Kalyuzhnaya et al., 2019). It is therefore possible the sediments samples had enough copper to put some methanotrophs (e.g., members of the family Beijerinckiaceae) that only possess sMMO in a competitive disadvantage, limiting their growth (Kalyuzhnaya et al., 2019). In any case, here we found more pMMO RNA transcripts attributed to CH_4_ oxidation in deeper coastal zones when compared to shallow areas. Considering that CH_4_ oxidation can account for 50–90% removal of CH_4_ before it escapes the sediment surface (King and Blackburn, 1996) this benthic CH_4_ oxidation might have a significant role in limiting CH_4_ escape.

### Methanotrophs and Limiting Abiotic Factors

In the study area, water depth strongly correlated with the increase in relative abundance of methanotrophs and RNA transcripts attributed to pMMO, with lower number of seabed CH_4_ seeps and CH_4_ concentrations in the surface water. We have not investigated if the seafloor geology influences this correlation as it would require a detailed geological mapping of the investigated area, including retrieval of longer sediment cores. However, abiotic factors that have been shown to affect methanotrophs and their activity along water depth are e.g., salinity (Osudar et al., 2017), oxygen and CH_4_ availability (King and Blackburn, 1996), ammonium concentrations (Bédard and Knowles, 1989; Murase and Sugimoto, 2005), and light (Murase and Sugimoto, 2005; Savvichev et al., 2019). At the time of sampling there was just minor changes in salinity in the studied stations (range: 6.5–7.0), compared to previous studies having a factor from freshwater-to-marine salinity (Bange, 2006; Osudar et al., 2015; Borges et al., 2016). Furthermore, a separate study conducted at the same time in the study area found no significant link between CH_4_ concentrations in the water and salinity [full details in Humborg et al. (2019)] indicating that salinity does not explain the patterns of relative abundance of methanotrophs and pMMO RNA transcript data in our study. The bottom water was oxygenated at all studied stations and was therefore unlikely to be a limiting factor for methanotrophs in the study area. In addition, the acoustic data showed more CH_4_ seeps in the inshore areas, and it is therefore unlikely CH_4_ production was a limiting factor in the sediment. However, in the current study CH_4_ concentrations were not measured in the sediment surface or bottom water, and such explanatory variables could help to further explain the findings in this study.

### The Effect of Ammonium on Methanotrophs in the Study Area

We measured higher pore water NH_4_^+^ concentrations in the offshore stations, and most of the measured NH_4_^+^ likely derived from organic matter mineralization in the suboxic and anoxic layers (Bonaglia et al., 2017) in and below the 0–2 cm sediment slices. It seems unlikely that the difference in pMMO transcripts between inshore and offshore is explained by methanotrophs actively oxidizing NH_4_^+^. For example, the number of RNA transcripts attributed to pMMO was on average 47 times higher than AMO in the offshore areas. Furthermore, the real-time CH_4_ measurements and acoustic data showed lower CH_4_ concentrations in the surface water and less seepages from the offshore sites. If methanotrophs were thriving on NH_4_^+^ more RNA transcripts for AMO than pMMO would be expected considering ammonia oxidizing bacteria oxidize NH_4_^+^ more effectively (Bodelier and Frenzel, 1999). Considering that ammonia oxidizing bacteria/archaea (You et al., 2009) had a significantly lower relative abundance in offshore sites, it is more likely that the availability of CH_4_ was driving growth of methanotrophs in the offshore stations. Moreover, in a laboratory experiment NH_4_^+^ concentrations below 36 mM have been observed to not influence methanotrophic activity (He et al., 2017) (our highest measurement was 0.4 mM NH_4_^+^). In addition, the NH_4_^+^ data does not explain why methanotrophs had a low relative abundance in the inshore sediments where NH_4_^+^ was also available. Furthermore, when data was analyzed together with PCA, most of variance for the offshore stations was explained by water depth, pMMO RNA transcripts, Methylococcales (16S rRNA gene%), and NH_4_^+^ concentrations (in this order, based on Pearson correlations between variables and principal components; Supplementary Table S8). For the inshore stations the variance was mainly explained by photosynthesis RNA transcripts and CH_4_ concentrations (Supplementary Data S8). Our results indicate that pore water NH_4_^+^ did not drive inhibition of methanotrophs or methanotrophic activity in the studied system.

### The Effect of Light on Methanotrophs in the Study Area

Considering that the geochemistry data (CH_4_ water concentrations and CH_4_ seabed seeps) and biological data (relative abundance of methanotrophs and RNA transcripts attributed to pMMO) both showed a relationship with water depth and that one of the main environmental factors changing along this gradient was light intensity, we suggest that illumination might influence sediment microbial communities. That the inshore stations were euphotic was indicated by (1) the CTD profiles showed that PAR light reached 28 m in the study area; (2) the detection of photosynthesis mRNA transcripts in the sediment (Figure 6B); and (3) benthic diatoms such as *Amphora* and *Nitzschia* (Vilbaste et al., 2000) in the 18S rRNA data (Supplementary Figure S8). Previous studies conducted in a reservoir and pelagic lake water have shown light to inhibit methanotrophy and increase CH_4_ water concentrations in northern South America and central Japan (Dumestre et al., 1999; Murase and Sugimoto, 2005). These studies included using bacterial cultures that would remove influencing factors on methanotrophy such as photosynthesis (i.e., producing oxygen) (King and Blackburn, 1996). Furthermore, Garcia et al. (2019) investigated the microbial community in a boreal lake with and without snow on the ice cover, and found that the relative abundance of methanotrophs decreased and CH_4_ water concentrations increased when the snow cover was removed and illumination increased in the water column. Additionally, the activity of NH_4_^+^ oxidizing bacteria are known to be inhibited by light availability (Guerrero and Jones, 1996). As the enzymes ammonia monooxygenase (AMO) and MMO are highly similar and evolutionary related (Holmes et al., 1995) it is possible that light availability plays a role in mediating sediment aerobic oxidation. However, additional studies are necessary to elucidate if there is such a relationship. Light has also been observed to stimulate methanotrophic activity in wetland sediments (Florida, United States) (King et al., 1990), and polar lake water (north-west Russia) while investigating bacterial cultures (Savvichev et al., 2019). These contrasting results in the literature could indicate that illumination affects various methanotrophic species differently or indirectly through other ecosystem processes. It is unknown if the low amount if light reaching the sediment at water depths 20–30 m in our study would have any effect on methanotrophs, and further work is needed to investigate if light has a negative effect on CH_4_ oxidation in shallow coastal ecosystems.

## Conclusion

The findings from this study are significant because natural aquatic environments are estimated to contribute to at least 30% of the global CH_4_ emissions (Dlugokencky Edward et al., 2011). For example, CH_4_ emissions from inland waters are known to significantly contribute to the atmospheric budget (estimated to 77 Tg C yr^–1^) (Bastviken et al., 2011). Humborg et al. (2019) calculated a daily sediment flux-water column CH_4_ reservoir of 2.5 mmol m^–2^ (or 30 mg C m^–2^) in the coastal waters of Tvärminne during September 2018 (same sampling campaign as reported in this study). This is within the range of CH_4_ emissions reported from subarctic lakes (Matveev et al., 2016), and it is suggested that shallow coastal waters, similarly to inland waters, are hotspots for CH_4_ emission. Moreover, limited methanotrophic activity could also help to explain why shallow coastal waters in rapidly changing ecosystems like the East Siberian artic shelf have higher CH_4_ emissions compared to the deeper offshore water (Shakhova et al., 2013; Thornton et al., 2016). Significant CH_4_ emissions from the artic subsea might therefore only occur in the shallowest parts due to ebullition and limited activity of methanotrophs in the sediment surface. Globally, low methanotrophic activity in the sediment surface, in addition to escaping CH_4_ bubbles, could explain the substantial amount of CH_4_ emissions from shallow inland water bodies and reservoirs (Deemer et al., 2016). This is an additional biological variable that can potentially contribute to explain the dynamics of greenhouse emissions from marine ecosystems. Future studies could investigate if sediment methanotrophs are limited by CH_4_ in shallow areas, and include more spatial variance with measured sediment characteristics (such as porosity and other abiotic variables).

## Author’s Note

This manuscript has been released as a pre-print at Research Square, Broman et al. (2020).

## Data Availability Statement

The datasets presented in this study can be found at: https://www.ncbi.nlm.nih.gov/, with accession numbers PRJNA541421 and PRJNA541422.

## Author Contributions

EB designed the study, sampled in the field, conducted laboratory work, bioinformatics, data analyses, and drafted the manuscript. XS measured CH_4_ during field sampling. CS collected acoustic data during field sampling and analyzed acoustic data together with MJ who compiled the results on maps. MS conducted RT-qPCR laboratory work and related data analysis. SB analyzed NH_4_^+^ samples in the laboratory. MG designed and developed the WEGAS system. AN sampled in the field. CH assisted with measurements of CH_4_ during field sampling. FN designed the study and contributed to drafting the manuscript. All authors gave feedback on the manuscript and gave final approval for publication.

## Conflict of Interest

The authors declare that the research was conducted in the absence of any commercial or financial relationships that could be construed as a potential conflict of interest.

## Footnotes

1 https://www.maanmittauslaitos.fi/en/node/1881
