# Supplementary material for: Low Abundance of Methanotrophs in Sediments of Shallow Boreal Coastal Zones With High Water Methane Concentrations
Source: Front Microbiol. 2020 Jul 7;11:1536. doi: 10.3389/fmicb.2020.01536 (PMC7362727; doi:10.3389/fmicb.2020.01536)
Supplement: Supplementary file 2 [file Table_2.DOCX]

**Low abundance of methanotrophs in sediments of shallow boreal coastal zones with high water methane concentrations**

Elias Broman, Xiaole Sun, Christian Stranne, Marco G. Salgado, Stefano Bonaglia, Marc Geibel, Martin Jakobsson, Alf Norkko, Christoph Humborg, Francisco J.A. Nascimento

**Supplementary Data**

**Data S1** The data shows the amount of sequences obtained after sequencing, quality filtering, extraction of 16S rRNA gene sequences, and results of the metagenome co-assembly.

**Data S2** Metagenome assembled *pmoA* and *pmoB* genes predicted with the software prodigal. The table includes information about prodigal contig, mapped RNA transcript counts per sample, results from BLAST classification against the UniProtKB/Swiss-Prot database, and the gene sequence. The orange coloured cells show which genes were used to design *pmoA* primers (as shown in Data S3).

**Data S3** Real-time qPCR results based on 16S rRNA gene primers and *pmoA* primers designed from the metagenome assembled *pmoA* genes (as shown in Data S3). The table shows CT values for each primer pair, the relative level RNA transcripts normalized for 16S rRNA transcript numbers (NRQ; normalized relative quantification) per sample, and the designed primer sequences. The primer names are based on the prodigal contig names (Data S2).

**Data S4** The data shows the lowest level of taxonomic annotation against the SILVA database annotation for the metagenome extracted 16S rRNA gene sequences. The values shown per sample are raw sequence counts.

**Data S5** The data shows the prokaryotic results from running Kraken2 against the NCBI RefSeq genome database using all sequences from the metagenomic DNA samples. The values shown per sample are raw sequence counts.

**Data S6** The data shows all results of the RNA transcripts (2018 RNA data) classified against the InterPro database. The values shown per sample are normalized sequence counts (CPM values) and raw sequence counts.

**Data S7** The data shows all results of the DNA functional genes (2017 and 2018 DNA data) classified against the InterPro database. The values shown per sample are normalized sequence counts (CPM values) and raw sequence counts.

**Data S8** The data shows the results from the PCA that was based on data from the September 2018 sampling cruise.


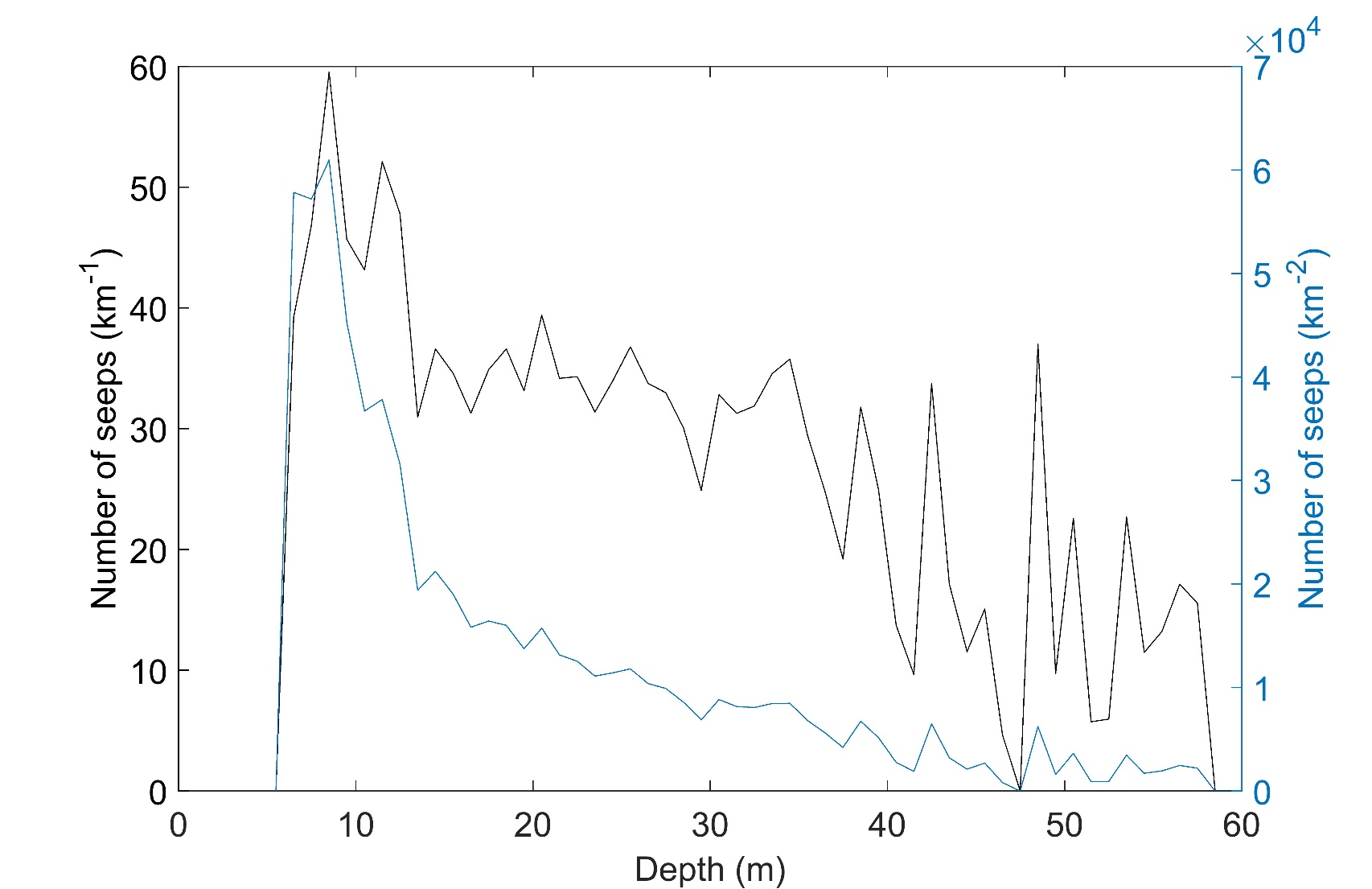


**Figure S1** Depth plotted against number of seeps per km (left y-axis) and against number of seeps per km2 (right y-axis).

**
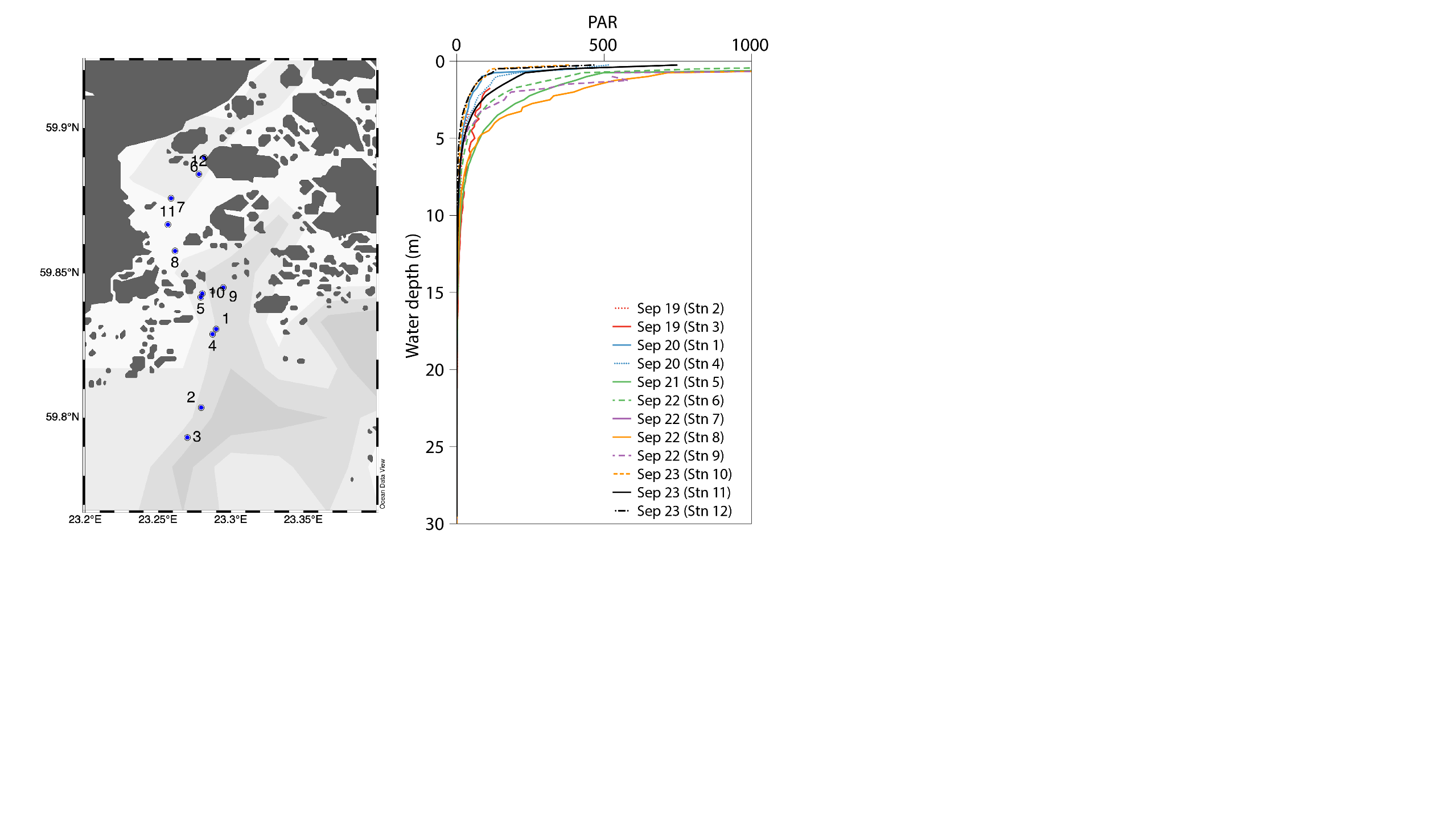
**

**
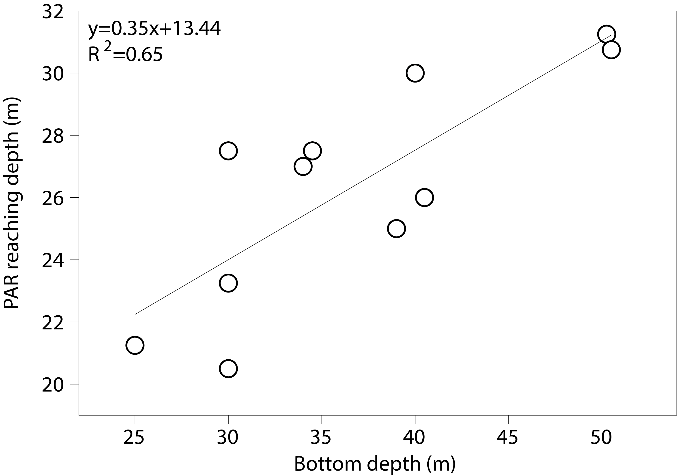

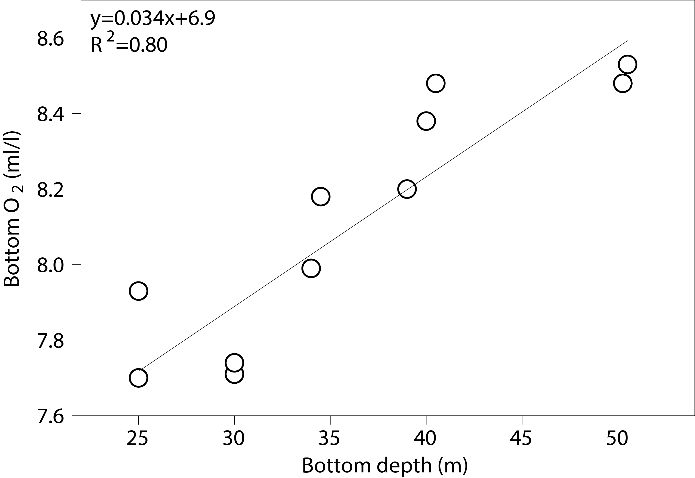
**

**Figure S2 Top panel:** CTD profiles from 12 locations inside the Storfjärden bay (note that CTD station numbers and not related to the station numbers were sediment was collected). PAR light profiles were collected during the 2018 sampling campaign between 19–23 September. **Bottom left panel:** Water depth vs the depth PAR is measured to be 0. **Bottom right panel:** Water depth vs oxygen concentrations in the bottom water. Station 5 is not shown in bottom panels as PAR never reached 0.

**
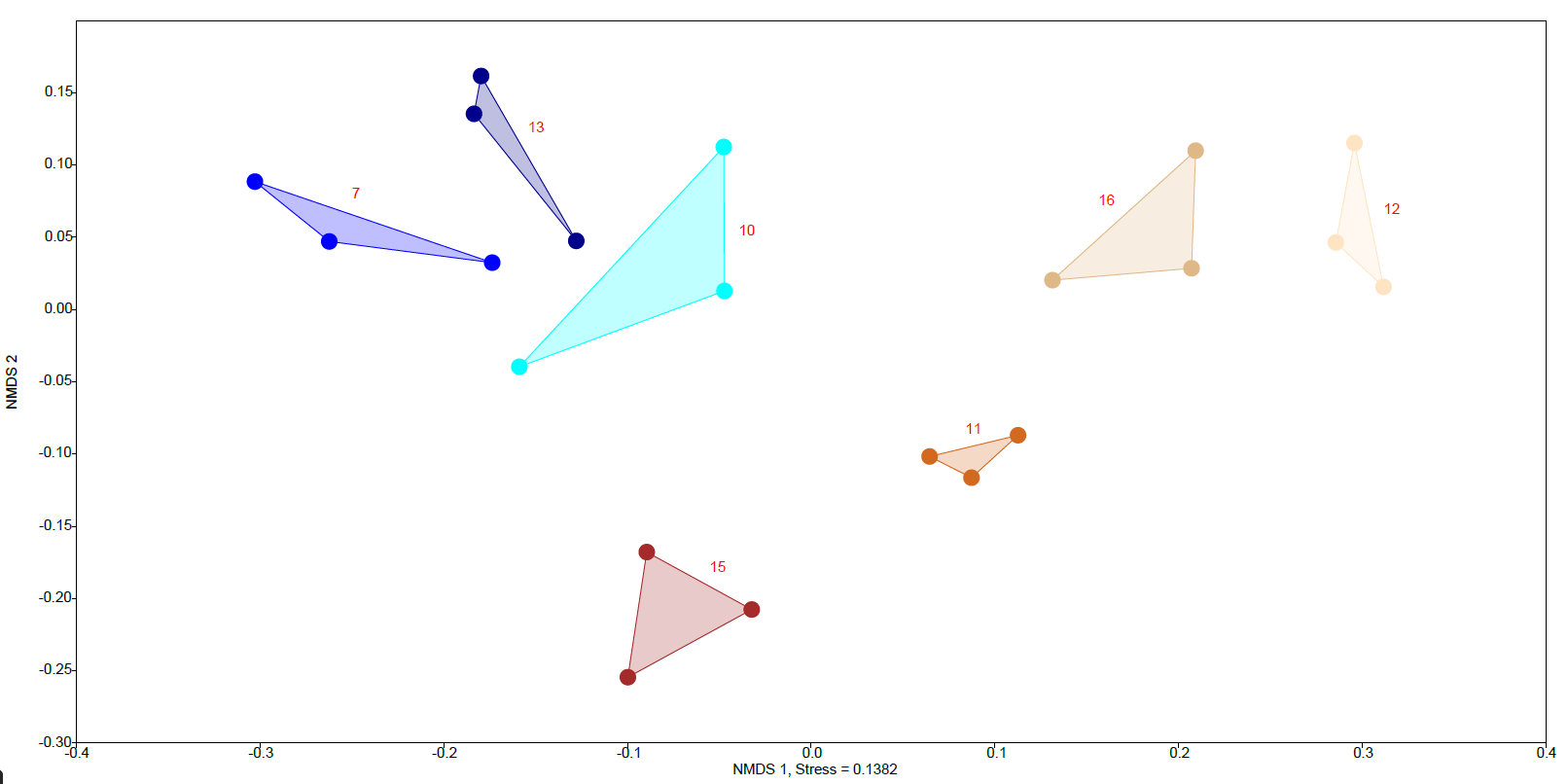
**

**Figure S3** NMDs showing the Bray-Curtis beta diversity of all samples (2018 16S rRNA gene DNA data). Station names are indicated next to their cluster of data points (*n* = 3 per station).


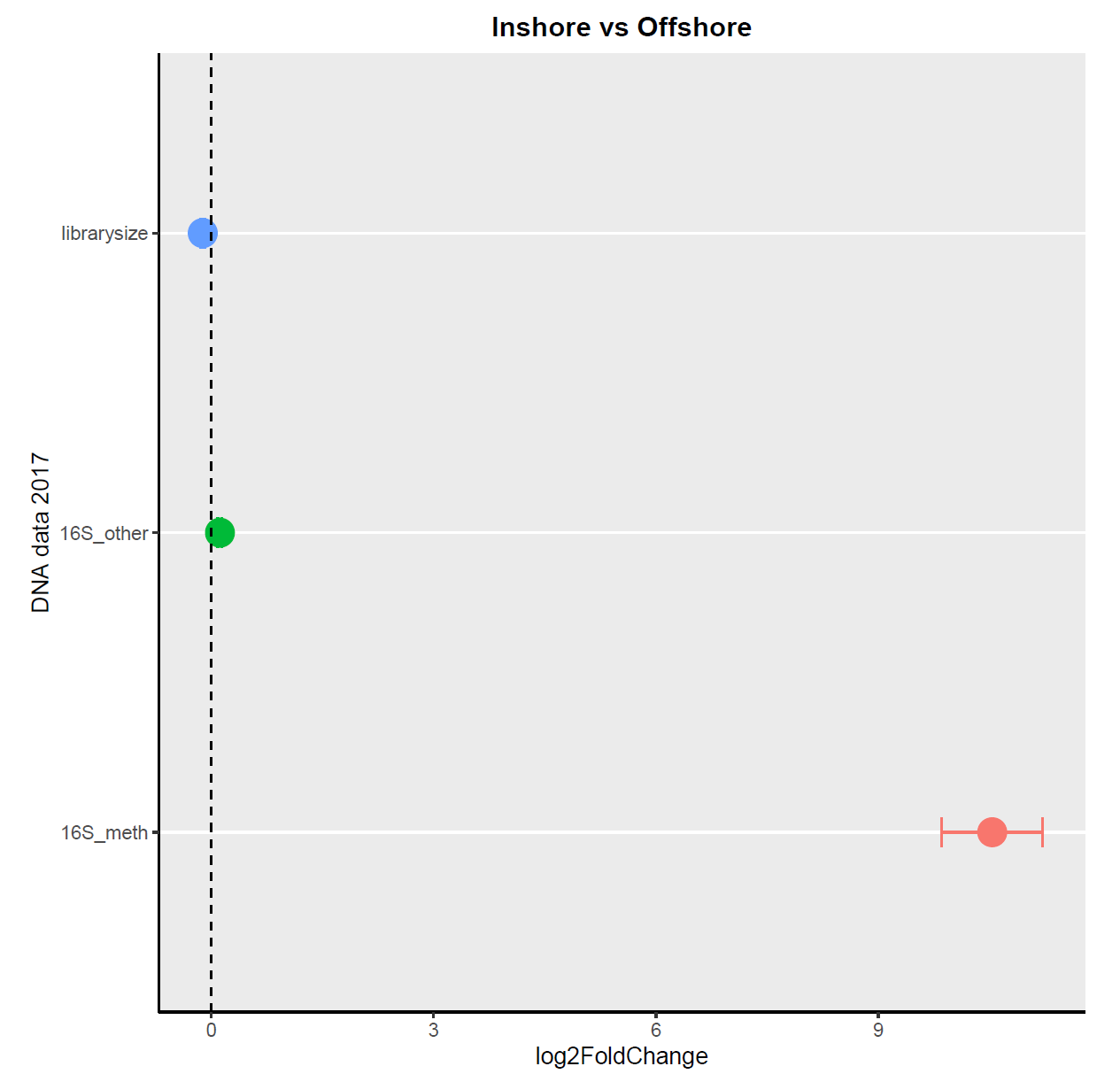


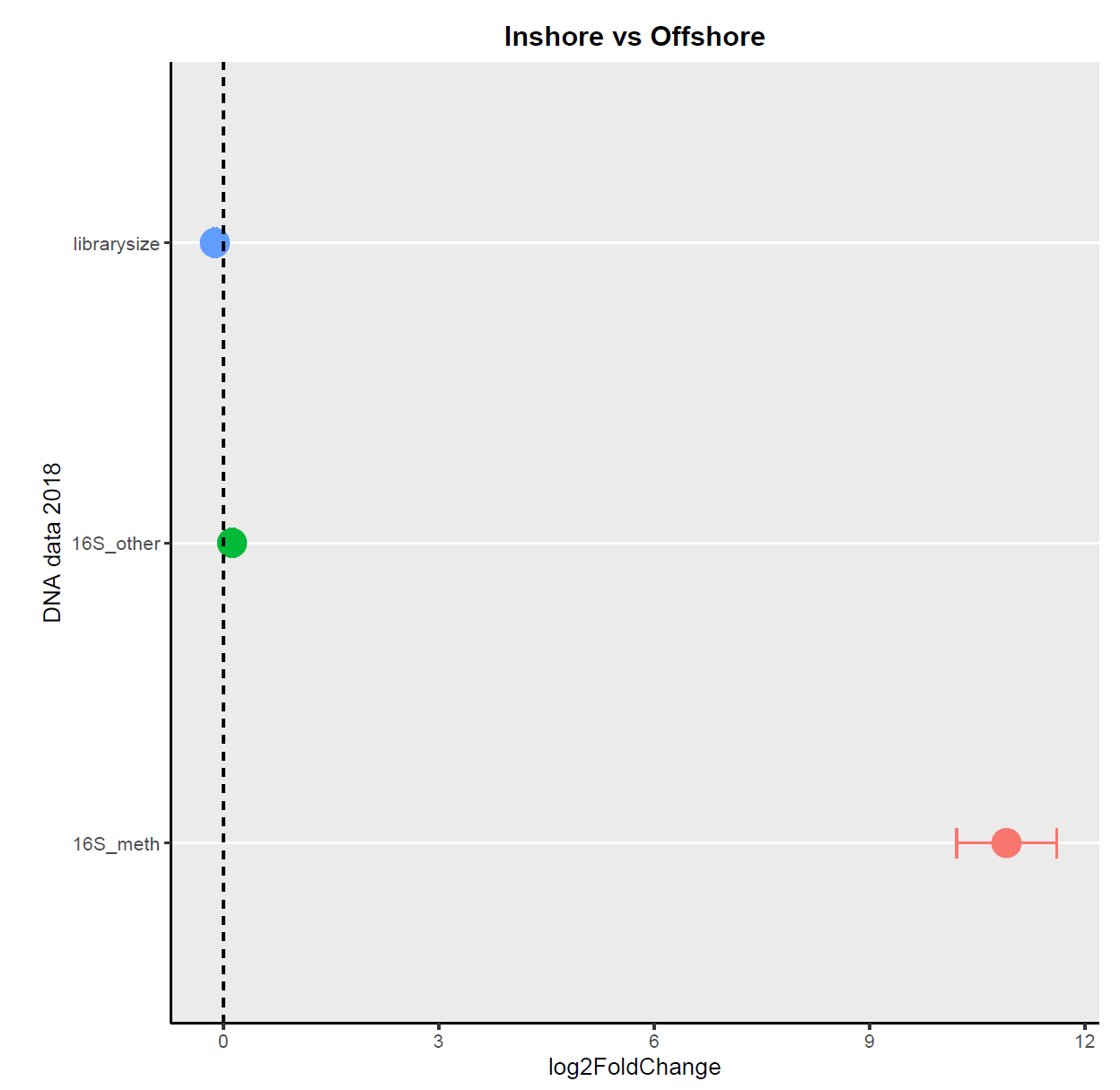


**Figure S4** Results from DESeq2 analysis testing the difference between inshore (left side of dashed line) and offshore stations (right side of dashed line). The data was based on counts attributed to *Methylococcales* 16S rRNA gene sequences (red, denoted as 16S_meth on y-axis), all other 16S rRNA gene sequences (green, denoted as 16S_other on y-axis), and the total library size of the DNA metagenome samples (blue). The top panel shows data for 2017, and the bottom panel shows data for 2018. The error bars show SE.

**
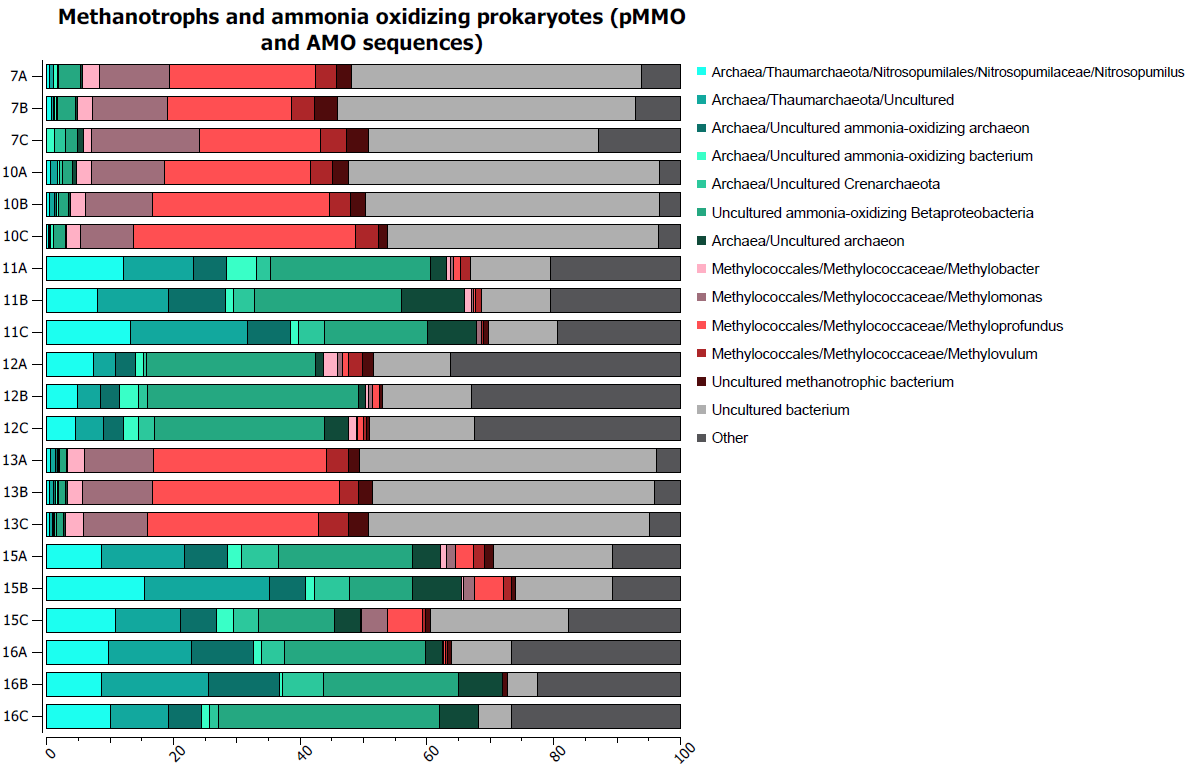
**

**Figure S5** The AMO and pMMO sequences were classified for taxonomy against NCBI NT using BLAST. The y-axis shows the sample stations, why the x-axis shows the relative abundance (%). Methanotrophs are coloured red in the figure, while ammonia oxidizers are coloured turquoise and green. “Other” denote taxonomic classifications <0.5%, and consisted mostly of ammonia oxidizing bacteria. The inshore stations consist of: 11, 12, 15, and 16; while the offshore stations consist of: 7, 10, and 13.

**
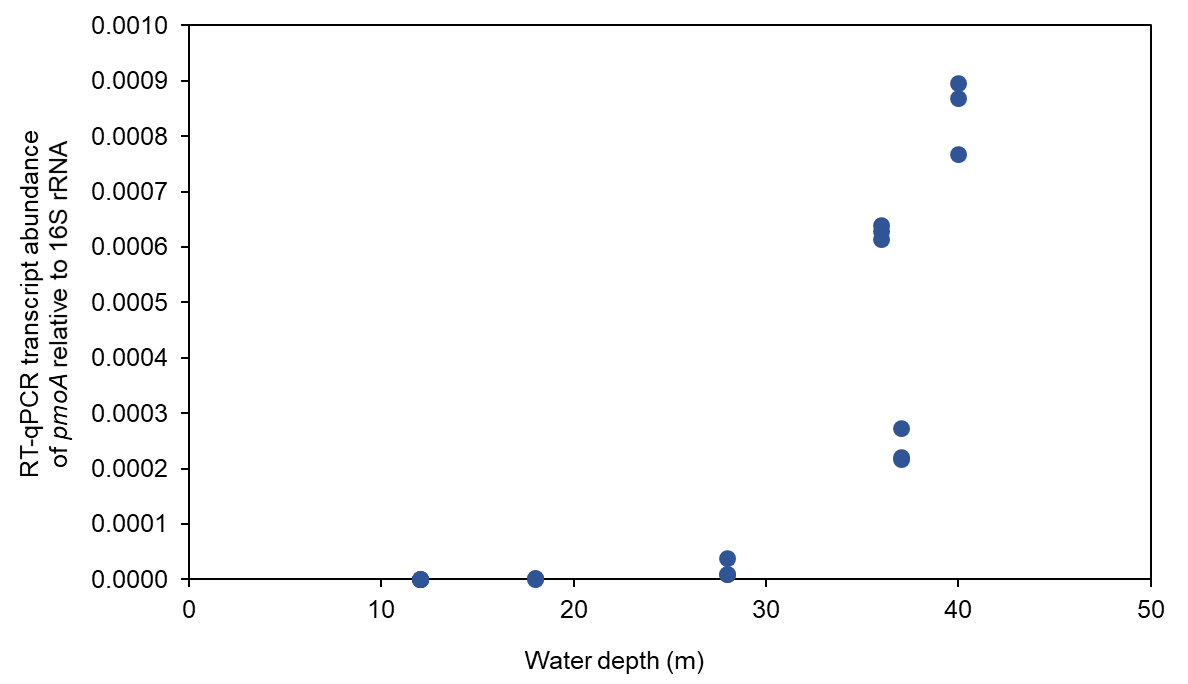
**

**
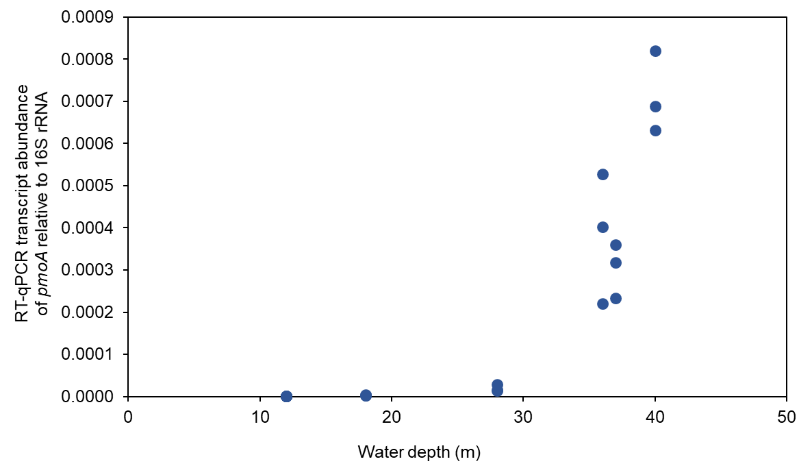
**

**Figure S6** RT-qPCR results showing the number of *pmoA* RNA transcripts relative to 16S rRNA for each station along the water depth gradient (y-axis shows NRQ; normalized relative quantification). The data is based on the designed *pmoA* primer pairs:

**Top panel**: pmoA_1700181_5_Fw - pmoA_1700181_5_Rv
**Bottom panel**: pmoA_13667928_2_Fw - pmoA_13667928_2_Rv

The primer names are based on the prodigal contig names (Data S2).


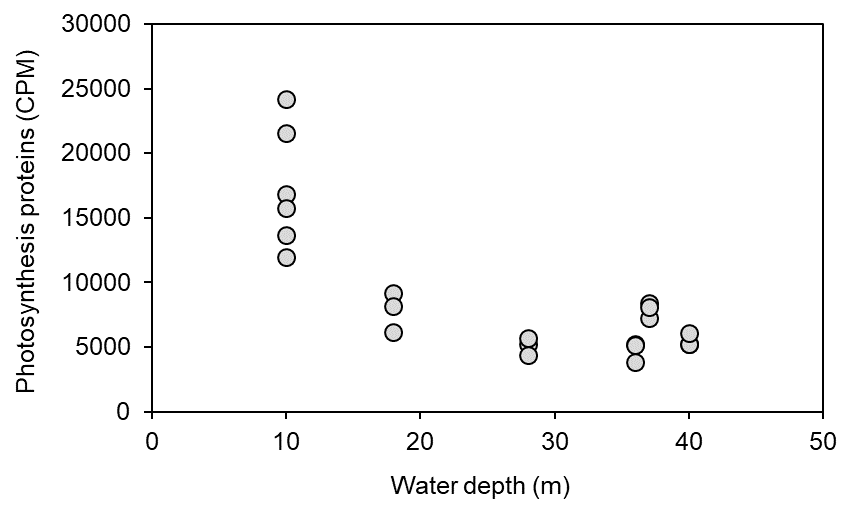


**Figure S7** The graph shows RNA transcripts attributed to proteins in the GO category photosynthesis (y-axis, CPM-values). The x-axis shows the water depth (m) in the studied stations.


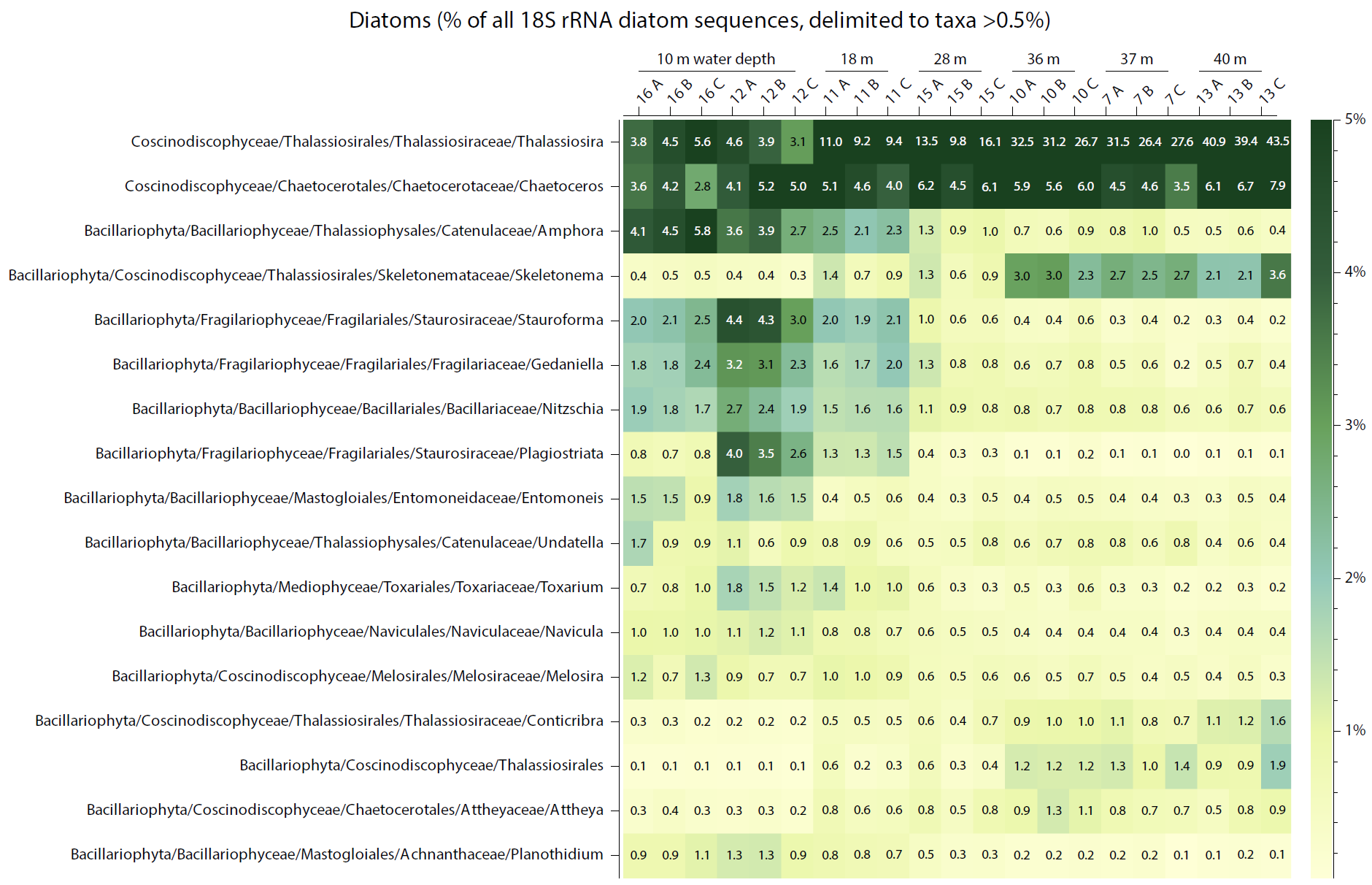


**Figure S8** Relative abundance of 18S rRNA sequences belonging to diatoms. The colour gradient and numbers in the heatmap shows the % per taxa compared to the whole diatom community. The heatmap only shows taxa with a total average > 0.5%.


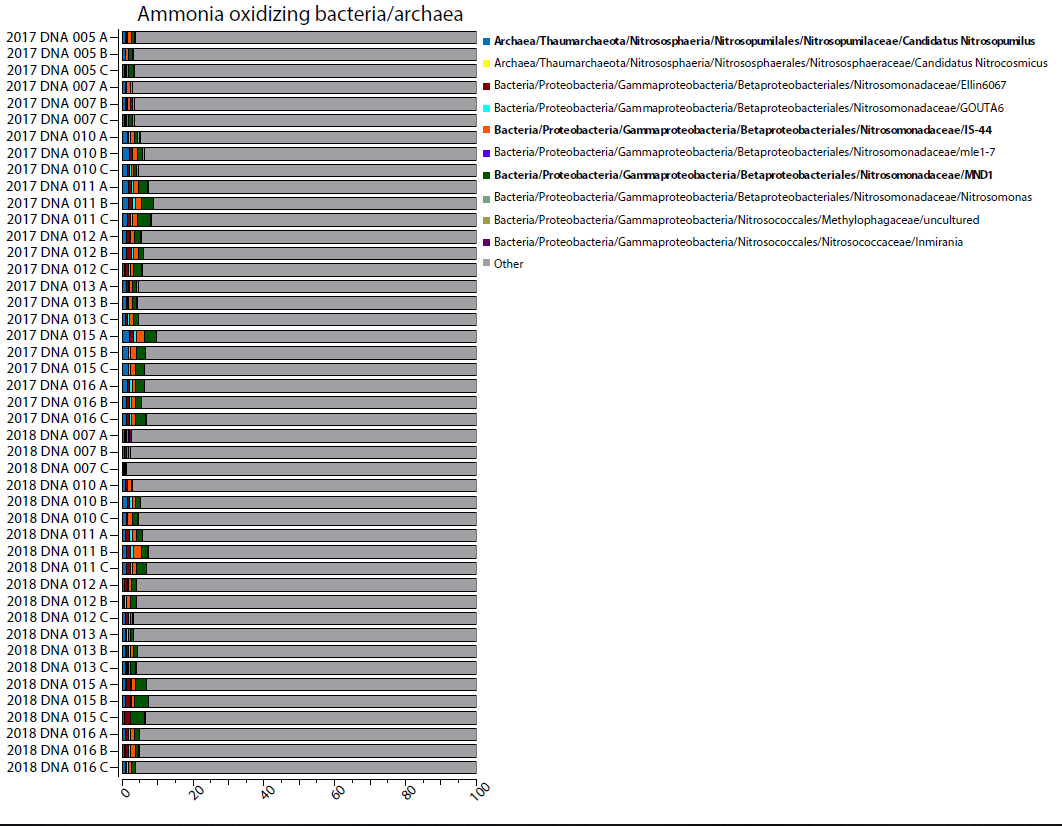


**Figure S9** The relative abundance of ammonia oxidizing bacteria. The x-axis shows the relative abundance (%) of the whole microbial community. Groups with a high relative abundance are indicated by bold text. The group “other” (denoted by grey colour) shows denotes all other prokaryotes.

**Table S1** CH_4_ measured in the 0–0.5 m surface water (nM), pore water NH_4_^+^ concentrations (µM), RNA transcripts attributed to proteins affiliated with the Photosynthesis GO category, whole microbial community AMO/pMMO proteins (CPM values), mapped RNA transcripts against metagenome assembled *pmoAB* genes (CPM), and RT-qPCR relative levels of *pmoA* transcript numbers (NRQ, normalized for 16S rRNA). Offshore stations are denoted with the label “off”, and dashes in the table denote no data collected.

| Station | **CH_4_**  2018 | **NH_4_^+^**  2018 | **Photosynthesis** | **InterPro AMO/pMMO** | | | **Mapped *pmoAB* transcripts** | **RT-qPCR *pmoA*** |
| --- | --- | --- | --- | --- | --- | --- | --- | --- |
|  |  |  | 2018  RNA | 2018 RNA | 2018 DNA | 2017 DNA | 2018  RNA | 2018  RNA |
| off-5 A | - |  | - | - | - | 1552 | - | - |
| off-5 B |  | - | - | - | - | 1536 | - | - |
| off-5 C |  |  | - | - | - | 1539 | - | - |
| off-7 A | 16.2 | 336 | 8402 | 6763 | 1455 | 1494 | 143 | 0.0020 |
| off-7 B |  | 417 | 7254 | 7250 | 1450 | 1485 | 195 | 0.0020 |
| off-7 C |  | 385 | 8131 | 9018 | 1445 | 1479 | 175 | 0.0023 |
| off-10 A | 15.8 | 297 | 5210 | 12 738 | 1460 | 1495 | 337 | 0.0037 |
| off-10 B |  | 267 | 5190 | 11 562 | 1528 | 1499 | 394 | 0.0033 |
| off-10 C |  | 251 | 3838 | 11 117 | 1486 | 1482 | 249 | 0.0028 |
| 11 A | 40.6 | 193 | 9187 | 1842 | 1519 | 1477 | 2 | 0 |
| 11 B |  | 199 | 8137 | 2156 | 1523 | 1429 | 5 | 0 |
| 11 C |  | 147 | 6128 | 2241 | 1500 | 1454 | 6 | 0 |
| 12 A | 29.8 | 194 | 16 818 | 1635 | 1458 | 1460 | 3 | 0 |
| 12 B |  | 161 | 15 792 | 1804 | 1457 | 1441 | 3 | 0 |
| 12 C |  | 170 | 13 684 | 2010 | 1502 | 1456 | 3 | 0 |
| off-13 A | 23.4 | 276 | 5247 | 12 530 | 1652 | 1459 | 503 | 0.0048 |
| off-13 B |  | 253 | 5222 | 12 990 | 1605 | 1488 | 491 | 0.0047 |
| off-13 C |  | 286 | 6100 | 10 165 | 1634 | 1504 | 290 | 0.0038 |
| 15 A | 23.4 | 281 | 5244 | 2709 | 1510 | 1533 | 13 | 0.0001 |
| 15 B |  | 283 | 4376 | 3285 | 1497 | 1503 | 22 | 0.0003 |
| 15 C |  | 254 | 5699 | 2577 | 1478 | 1548 | 15 | 0.0001 |
| 16 A | 23.6 | 152 | 21 523 | 1855 | 1491 | 1477 | 1 | 0 |
| 16 B |  | 156 | 24 214 | 1606 | 1494 | 1500 | 0 | 0 |
| 16 C |  | 167 | 11 963 | 1674 | 1482 | 1453 | 0 | 0 |
